# Cervicoscopy and Microcolposcopy in the Evaluation of Squamo Columnar Junction and Cervical Canal in LSIL Patients with Inadequate or Negative Colposcopy

**Published:** 2013-09

**Authors:** Edoardo Valli, Guido Fabbri, Chiara Centonze, Alessandro Bompiani, Federico Baiocco, Giovanni Larciprete, Alessio Ghinassi

**Affiliations:** 1 Tor Vergata University, Rome Italy;; 2 Department of Obstetrics and gynecology, Fatebenefratelli Hospital, Isola tiberina Rome, Italy;; 3 L’altrastatistica srl - Consultancy & Training - Biostatistics office, Italy

**Keywords:** cervicoscopy, microcolposcopy, inadequate colposcopy, squamocolumnar junction, LSIL

## Abstract

**Objective:**

The present study evaluated indications’ validity of cervicoscopic and microcolposcopic examination in LSIL patients with unsatisfactory or negative colposcopy.

**Matherial and methods:**

In the cervico-vaginal pathology unit of the “San Giovanni Calibita Fatebenefratelli” University of Rome “Tor Vergata”, 119 patients with a positive cervical cytology (LSIL), were submitted to the exam for the following two indications: 1) unsatisfactory colposcopy 37 (31.1%); 2) negative colposcopy 82 (68.9%).

**Results:**

Cervicoscopy allowed the SCJ visualization in 115 (9.6%) patients. In 4 patients 3.4%, the SCJ visualization was not possible due to cervical stenosis. Cervicoscopy without staining, revealed endocervical squamous columnar junction in 33 (28.7%) patients. The blue dye in panoramic view detected endocervical SCJ in 41 (35.7%), out of 115 patients (>5 mm in 34 (29.6%) patients and >10 mm in 7 (6.1%)).

**Conclusions:**

Cervicoscopic examination revealed 7.8% of CIN2-3 in LSIL patients with inadequate or negative colposcopy. In patients with negative colposcopy the percentage of undiagnosed lesions inside the cervical canal was very low. The blue dye added sensitivity to the exam.

## INTRODUCTION

The better knowledge of the evolution of the cervical disease, concerning timing and modalities of progression, allows to follow up a high number of low grade lesions without treatment (1, 2). The “see and wait” policy requires the complete mapping of the lesion, and the evaluation of the HPV DNA or of the p16 immunostaining (3-5). Colposcopic examination allows to evaluate lesions and their progression or regression (6-10) but, it has its own limitation when the examination is unsatisfactory (or inadequate, according to the new colposcopic terminology), that is when the squamocolumnar junction (SCJ) is not completely evaluable or the lesion extends inside the cervical canal (11). A different and frequent condition is the adequate and negative colposcopy in patients with abnormal cytology. Many authors consider appropriate, under these conditions, the evaluation of the cervical canal, utilizing the technique of the endocervical curettage. Endocervical curettage (ECC) is a blind and painful technique with low sensitivity because often the tissue obtained is not sufficient for a reliable histological diagnosis (14-18). Hamou (12) proposed the Microcolposcopy carried out with a dedicate instrument as a diagnostic method for evaluation of cervix and cervical canal, but this exam is now rarely employed. The availability of new instrumentation and video recording allows to evaluate the cervical canal utilizing a standard office hysteroscopic instrument (cervicoscopy) (13). For these reasons the present study evaluates the indications and results of cervicoscopic and microcolposcopic examination of SCJ and of cervical canal in LSIL patients.

## MATERIALS AND METHODS

In the cervico-vaginal pathology unit of the “San Giovanni Calibita Fatebenefratelli” part of the Division of Perinatal Medicine, University of Rome “Tor Vergata”, we performed, from January 2009 until June 2011, 290 microcolposcopy examinations.

Among these we retrospectively selected, for the purpose of the study, 119 patients with a positive cervical citology (LSIL), who underwent the microcolposcopy for the following two indications:

1. Inadequate colposcopy 37 (31.1%);
2. Negative colposcopy 82 (68.9%).

All the patients signed a written informed consent before the exam.

The examinations were conducted by a single operator (E.V.), using a 2.9 Bettocchi hysteroscope (Storz) with a 4 mm single-flow diagnostics sheath for the infusion of saline solution, which allows the continuous distension and cleaning of the cervix. The staining of squamous and metaplastic cells was obtained with Waterman blue (a non-toxic dye introduced and tested for the absence of toxicity and / or adverse reactions by Hamou) (12) which was applied with a sterile swab at eso and endocervical level. The examination was conducted following 3 steps.

**Figure 1:**
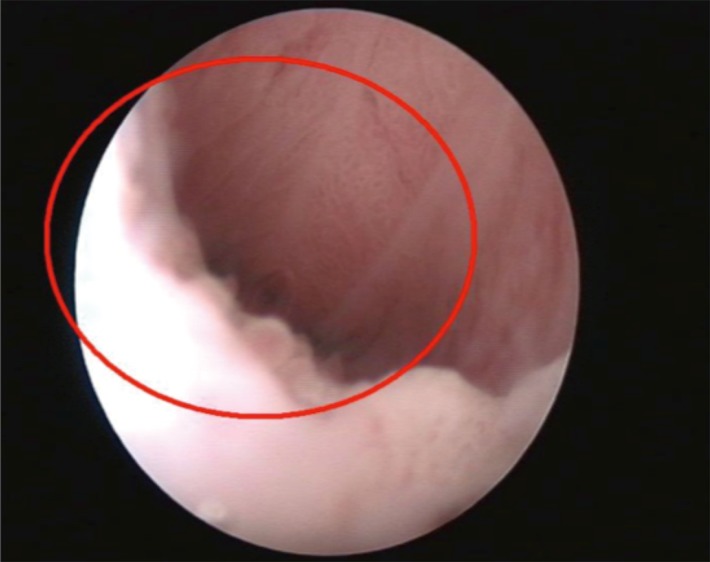
Evaluation of SCJ with saline solution.

The first step (cervicoscopy) (Fig. 1) consisted of the introduction of the instrument in the cervical canal only with the saline solution distension for the evaluation of SCJ. The SCJ was reported as esocervical, at the external uterine orifice (EUO) or endocervical if >5 mm from the external cervical orifice. The cervical canal was evaluated until the internal uterine orifice (IUO). The uterine cavity was evaluated performing a complete hysteroscopic examination only for further indications or pathology widespread until the IUO.

**Figure 2:**
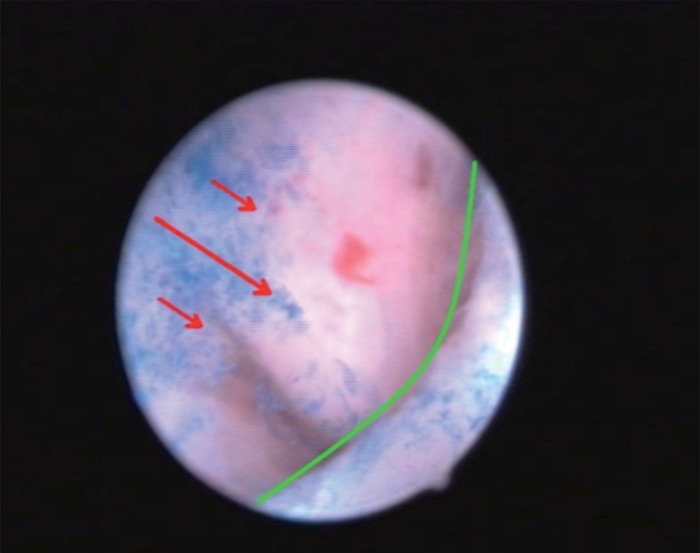
SCJ with blue die in panoramic view.

The second step consisted of the staining with the Waterman blue dye and of the examination of the SCJ in panoramic view in order to highlight areas of staining in the cervical canal indicative for the presence of metaplastic or squamous cells at this level (Fig. 2).

The third step consisted of the contact examination for the evaluation of cellular aspects (microcolposcopy).

**Figure 3:**
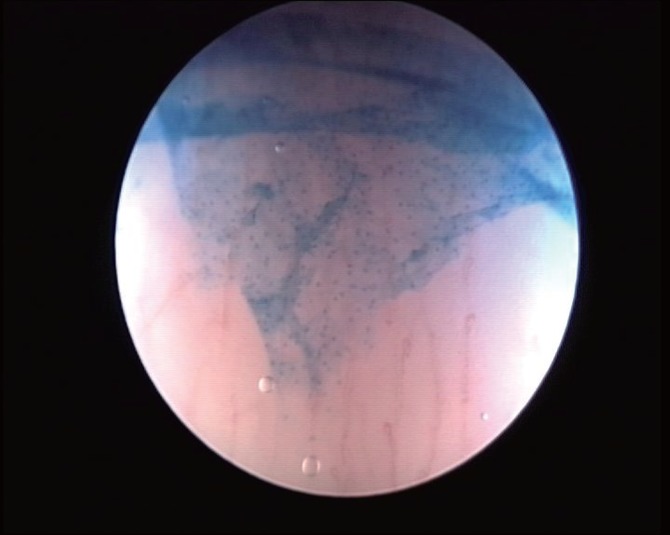
Contact view: normal superficial cells (G0).

**Figure 4:**
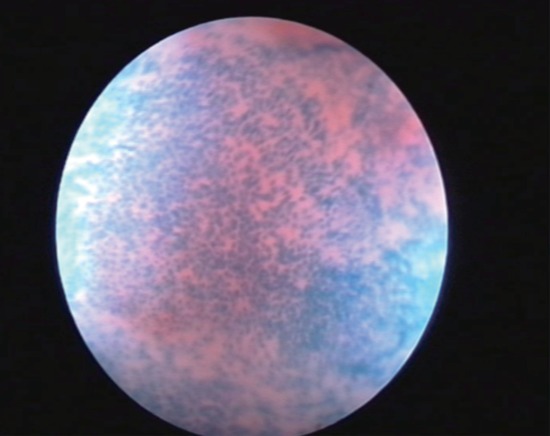
Mild nuclear volume increasing: (G1).

**Figure 5:**
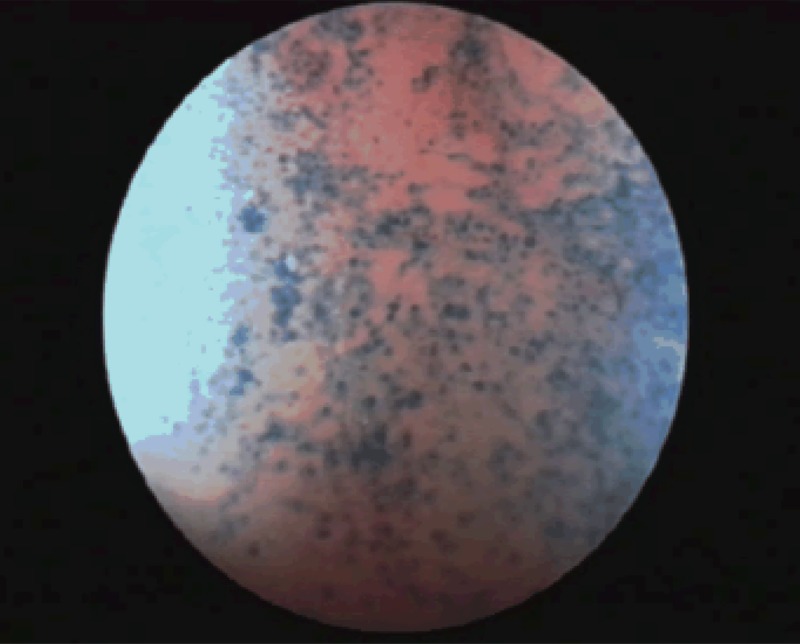
High grade nuclear abnormalities (G2).

The evaluation of cellular abnormalities was graded according to the classification proposed by Hamou (12), which consists of evaluating the size and homogeneity of the superficial squamous cells. These cells were classified as: normal or grade 0 (G0) when superficial cells showed regular and picnotic nuclei with a regular nucleocytoplasmatic proportion (Fig. 3), grade 1 (G1) when nuclei mildly increased in volume with uniform size were present (Fig. 4) and grade 2 (G2) in the presence of greatly increased and disomogeneous nuclei with an altered nucleocytoplasmatic proportion (Fig. 5). The cellular alterations were mapped and if located inside the cervical canal, their depth was evaluated measuring the distance from the apex of the lesion to the EUO using the microcolposcope’s sheath. When cellular abnormalities were found, a targeted biopsy was performed. Negative patients were invited to a cytologic and colposcopic follow up at 6 months. Histological results were compared with the microcolposcopy. Statistical analisys of results was performed using Chi-squared and student T-test.

## RESULTS

Results were divided considering the 3 steps of the examination. The cervicoscopy allowed the SCJ visualization in 115 (96.6%) patients.

In 4 patients (3.4%) the SCJ visualization was not possible because of EUO stenosis caused by previous cold knife conization.

**Figure 6:**
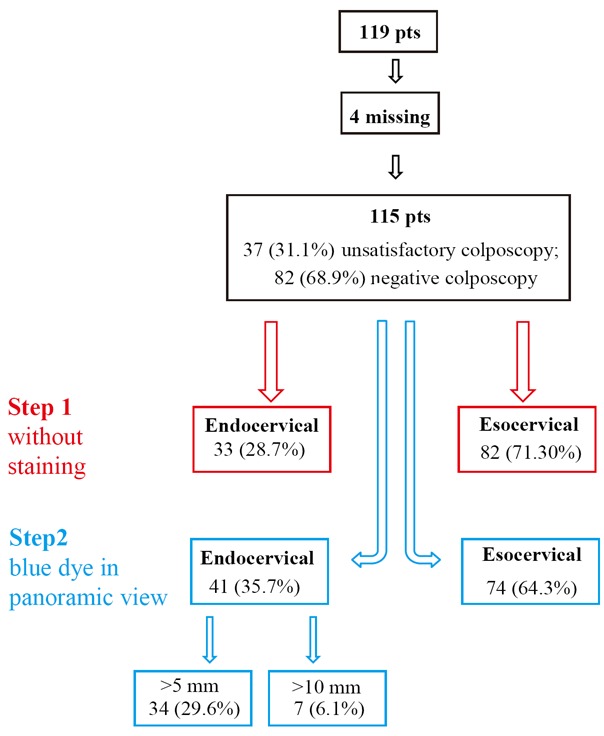
Cervicoscopic steps for the evaluation of SCJ.

The SCJ without staining resulted endocervical in 33 (28.7%) patients, and the blue dye detected the SCJ endocervical in 41 (35.7%), >5 mm in 34 (29.6%) and >10 mm in 7 (6.1%) (Fig. 6).

Endocervical SCJ was detected in 18/33 (54.5%) patients from the unsatisfactory colposcopy group, and in 23/82 (28.0%) patients with negative colposcopy (*p*-value 0.007, Table 1).

**Table 1:** SCJ position

INDICATION	UNSATISFACT. COLPOSCOPY		NEGATIVE COLPOSCOPY	

	**n**	**%**	**n**	**%**
**SCJ**				
**ESOCERV.**	15	45.5%	59	72%
**ENDOCERV.**	18	54.5%	23	28%
**TOTAL**	33		82	

Contact examination of the SCJ (Table 2), detected G1 cellular alterations in 28 (24.3%) patients and G2 in 25 (21.7%).

**Table 2:** Results of contact microcolposcopy

	G0		G1		G2		TOTAL
	n	%	n	%	n	%	

NEGATIVE COLPOSCOPY	55	67.1%	13	15.9%	14	17.1%	82
UNSATISF. COLPOSCOPY	7	21.2%	15	45.5%	11	33.3%	33

G2 cellular abnormalities were detected in 11 (33.3%) of the patients with inadequate colposcopy, and in 14 (17.1%) with negative colposcopy (*p*-value 0.079).

Histological results showed in 19 patients (35.84% of biopsies), the presence of intraepithelial neoplasia (10 CIN 1; 9 CIN 2-3); others were metaplastic inflammatory or atrophic changes.

## DISCUSSION

The evaluation of cervical canal has been performed with many different methods and for different indications. Many authors propose endocervical curettage (14-18), that is a blind and painful technique, with a high percentage of false negative, while the hysteroscopic evaluation of cervical canal is rarely reported in literature (13). Our results conversely show that cervicoscopy can evaluate the SCJ almost in every case, with the only exception of post surgical stenosis not allowing the overcoming in the cervical canal. Our evaluation showed a higher percentage of endocervical SCJ with the blue dye application. The difference can be due to the fact that, blue dye indicates the presence of metaplastic endocervical cells that were not recognisable without coloration. The results also show a significant difference in the percentage of endocervical SCJ with respect to the indication of the exam, being higher when colposcopy was unsatisfactory.

Finally, our results show that cervicoscopy identified in LSIL patients with inadequate colposcopy a percentage of 7.8% patients with high grade lesions, otherwise considered negative, and conversely avoided a blind endocervical curettage in the great majority of these patients. The blue dye added sensitivity to the exam and it can be employed in panoramic view without a specific cytologic experience. Microcolposcopy with contact examination resulted to be useful for the prevision of results, grading the lesion with a higher sensitivity, but it requires a specific training.

In conclusion, we propose the introduction of cervicoscopy in LSIL patients with inadequate colposcopy in the work up for the prevention of cervical cancer.

Further research on endocervical targeted biopsies performed also in negative patients should better establish the negative predictive value of the exam, in order to perform targeted biopsies only in G2 patients, and completely avoiding the endocervical curettage.
